# Anti-staphylococcal activity of soilless cultivated cannabis across the whole vegetation cycle under various nutritional treatments in relation to cannabinoid content

**DOI:** 10.1038/s41598-024-54805-3

**Published:** 2024-02-22

**Authors:** Lucie Malikova, Matej Malik, Jan Pavlik, Milos Ulman, Eva Pechouckova, Milos Skrivan, Ladislav Kokoska, Pavel Tlustos

**Affiliations:** 1 Department of Microbiology, Nutrition and Dietetics, Faculty of Agrobiology, Food and Natural Resources, Czech University of Life Sciences Prague, 165 00 Prague-Suchdol, Czech Republic; 2 Department of Nutritional Physiology and Animal Product Quality, Institute of Animal Science, 104 00 Prague-Uhrineves, Czech Republic; 3 Department of Agroenvironmental Chemistry and Plant Nutrition, Faculty of Agrobiology, Food and Natural Resources, Czech University of Life Sciences Prague, 165 00 Prague-Suchdol, Czech Republic; 4 Department of Information Technologies, Faculty of Economics and Management, Czech University of Life Sciences Prague, 165 00 Prague-Suchdol, Czech Republic; 5 Department of Crop Science and Agroforestry, Faculty of Tropical AgriSciences, Czech University of Life Sciences Prague, 165 00 Prague-Suchdol, Czech Republic

**Keywords:** Antimicrobials, Pathogens

## Abstract

Antibiotic resistance in staphylococcal strains and its impact on public health and agriculture are global problems. The development of new anti-staphylococcal agents is an effective strategy for addressing the increasing incidence of bacterial resistance. In this study, ethanolic extracts of *Cannabis sativa* L. made from plant parts harvested during the whole vegetation cycle under various nutritional treatments were assessed for in vitro anti-staphylococcal effects. The results showed that all the cannabis extracts tested exhibited a certain degree of growth inhibition against bacterial strains of *Staphylococcus aureus*, including antibiotic-resistant and antibiotic-sensitive forms. The highest antibacterial activity of the extracts was observed from the 5th to the 13th week of plant growth across all the nutritional treatments tested, with minimum inhibitory concentrations ranging from 32 to 64 µg/mL. Using HPLC, Δ^9^-tetrahydrocannabinolic acid (THCA) was identified as the most abundant cannabinoid in the ethanolic extracts. A homolog of THCA, tetrahydrocannabivarinic acid (THCVA), reduced bacterial growth by 74%. These findings suggest that the cannabis extracts tested in this study can be used for the development of new anti-staphylococcal compounds with improved efficacy.

## Introduction

Despite the prevalence of literature characterizing staphylococcal pathogenesis in humans, *Staphylococcus aureus* is a major cause of infections and diseases in a plethora of animal hosts, leading to a considerable impact on public health and agriculture^1^. As a highly adaptive pathogen, this bacterium is responsible for a wide range of clinical problems, from minor skin infections to life-threatening bacteremia and meningitis^2^. Serious infections associated with *S. aureus* cause approximately 20,000 human deaths annually in the US and 5000 in the EU, costing an estimated 380 million € in health costs^3^. While penicillins were historically effective against *S. aureus*^4^, the use of conventional antibiotics as a standard treatment of staphylococcal infections has become problematic due to their serious side effects^5^ and the emergence of drug-resistant *S. aureus* strains^1^. Currently, the global spread of methicillin-resistant *S. aureus* (MRSA) is one of the most serious public health challenges because MRSA strains have emerged with concomitant resistance to other groups of antibiotics, such as aminoglycosides, macrolides, and tetracyclines as well as the beta-lactam antibiotics^6^. The rise of antibiotic resistance poses a growing threat to bacterial infection treatment^7^, with MRSA strains significantly contributing to morbidity and mortality compared to methicillin-sensitive *S. aureus* (MSSA) (15.6% vs. 6.2%)^8^. The development of new anti-staphylococcal agents, encompassing the use of novel molecules and natural extracts as innovative therapeutics, is an effective strategy to address growing bacterial resistance^9,10^, and innovative approaches to medicinal plant cultivation play a crucial role in this endeavor^11^.

Recognizing the escalating threat of antibiotic-resistant strains, including those of *S. aureus*, cultivators of medicinal plants for pharmaceutical and healthcare purposes are turning to controlled indoor facilities for cultivation. These environments mitigate risks associated with unpredictable outdoor conditions and pests, allowing for the production of homogeneous plant material^12,13^. The choice of cultivation substrate is critical, involving soil-based or soilless media (hydroponics)^14^. Soilless culture systems, defined as growing plants without using soil as a rooting medium while supplying inorganic nutrients via irrigation nutrient solution^15,16^, offer cost-effectiveness, higher yields, and prompt harvests^17,18^. Soilless systems also generally have higher water and nutrient use efficiencies^19,20^. A proper supply of mineral nutrients is essential for efficient and sustainable cultivation^21^. Notably, essential nutrients such as nitrogen (N), phosphorus (P), and potassium (K) are pivotal in regulating the profile of secondary metabolites in plants^22,23^. Although emphasis is placed on the availability of sufficient amounts of these major plant nutrients, the potential effects of micronutrients^24^ and plant biostimulants^25^ must be considered. Iron (Fe), for instance, is indispensable for fundamental plant processes such as photosynthesis and respiration^26^. Furthermore, the integration of plant biostimulants, particularly amino acids, is gaining recognition for their role in modulating nitrogen absorption and assimilation by regulating the enzymes and structural proteins involved in these processes^27^. While the effects of essential nutrients and biostimulants on plant metabolism are a significant area of many studies^28–30^, further research is needed to fully understand their impact on the formation of secondary plant metabolites.

Among the various crop species grown in soilless systems, such as spices^31^ and culinary aromatic herbs^32^, medical cannabis (*Cannabis sativa* L.) is commonly cultivated to control nutrient delivery and environmental conditions for optimal bioactive compound production^33^. The acceptability, utilization, and subsequent medicinal use of *C. sativa* continue to expand, as shown by the growing number of countries that allow its use for specific therapeutic purposes^34–36^. The beneficial potential of cannabis can be attributed to its rich metabolite profile, with more than 480 compounds detected^37^, particularly to cannabinoids such as cannabidiol (CBD), cannabigerol (CBG), and Δ^9^-tetrahydrocannabinol (Δ^9^-THC)^38^. The diverse pharmacological profile of cannabis includes anticancer or anti-inflammatory effects^39^ and, notably, antimicrobial activity against various pathogenic bacteria such as *S. aureus* (including MRSA), *Streptococcus mutans,* or *Listeria monocytogenes*^40,41^. The ability of crude cannabis extracts and phytocannabinoids to curtail the growth and spread of *S. aureus* has been the subject of many studies^1,37^ since the pioneering work by Van Klingeren and Ten Ham in 1976^42^. Their study highlighted the anti-staphylococcal effects of phytocannabinoids, with CBD and Δ^9^-THC significantly affecting the growth of *S. aureus*. Furthermore, extracts from *C. sativa* and phytocannabinoids have demonstrated the ability to inhibit the synthesis of bacterial virulence factors and the formation of biofilms^43^, providing a potential solution to the challenges posed by limited sensitivity to antibiotics^44,45^. However, the antibacterial activity of these compounds depends on various factors, including the specific phytocannabinoid compound being used and its concentration^46^. Considering the strong antibacterial potential of cannabis extracts, this study evaluated the growth-inhibitory effect of ethanolic extracts of *C. sativa* L. against two *S. aureus* strains, including the antibiotic-resistant form, across the vegetation cycle to identify cannabinoids with the highest antimicrobial activity and to determine the optimal harvest time in the context of soilless cultivation.

## Results

### Phytocannabinoid content in ethanolic cannabis extracts

The concentration of cannabinoids in the ethanolic cannabis extracts was measured for all the individual treatments (A, B, C, and D) every week throughout the entire vegetation cycle (13 weeks). Seventeen phytocannabinoids were identified and quantified using standard curves. The eight cannabinoids with the highest concentration and most stable occurrence across the vegetation cycle of harvested plants cultivated under different treatments are described in Table 1. As in cannabis chemical phenotype I, the most concentrated cannabinoid in the plants and consequently also in the extracts was Δ^9^-tetrahydrocannabinolic acid (THCA). The THCA content in the plants increased with time, and its maximum concentration was reached at full maturity in the 11th week. The plants grown in treatment D ripened a week earlier, in the 10th week, but they also achieved the highest average concentration of THCA in the cannabis extract (1915 ± 39 μg/mL). The concentration of the decarboxylation products of THCA and Δ^9^-THC and the oxidative degradation product of THCA, cannabinolic acid (CBNA), increased proportionally with the THCA content. A similar trend was shown for the cannabivarin homolog of THCA, tetrahydrocannabivarinic acid (THCVA), which has a propyl side chain instead of a pentyl, the biosynthetic precursor of THCA, cannabigerolic acid (CBGA), and cannabidiolic acid (CBDA). The concentration of CBCA gradually decreased in proportion to the age of the plant. The other phytocannabinoids showed no consistent trend over time.

**Table 1:** Cannabinoid concentration (µg/mL) in cannabis extracts from different treatments by vegetation week.

Treatment	Week	Phytocannabinoids							
		CBDVA	CBDA	CBGA	THCVA	CBNA	Δ^9^-THC	THCA	CBCA
All^a^	1	0.3 ± 0.1	1.6 ± 0.6	5.4 ± 0.6	1.3 ± 0.2	0.2 ± 0.2	7.7 ± 1.7	226 ± 44	88.9 ± 7.3
A^b^, B^c^	2	0.2 ± 0.1	1.2 ± 0.2	8.4 ± 1.9	1.3 ± 0.4	0.2 ± 0.1	7.5 ± 1.4	232 ± 34	74.4 ± 8.6
C^d^, D^e^		0.1 ± 0.1	1.4 ± 0.1	12.2 ± 2.2	1.3 ± 0.3	0.3 ± 0	9.2 ± 3	236 ± 44	37.7 ± 1.1
A^b^, B^c^	3	0.5 ± 0.1	1.4 ± 0.2	8.7 ± 0.5	1.2 ± 0.2	0.6 ± 0.1	9.9 ± 3	232 ± 31	62.2 ± 10.7
C^d^, D^e^		0.2 ± 0.1	1.6 ± 0.3	8.5 ± 0.2	1.2 ± 0.1	0.5 ± 0.2	9.6 ± 2.2	265 ± 22	51.4 ± 2.3
A^b^, B^c^	4	0.5 ± 0.1	1.4 ± 0	11.2 ± 1.8	1.2 ± 0.2	0.7 ± 0.3	17.7 ± 1.8	286 ± 24	44.8 ± 6.8
C^d^, D^e^		0.1 ± 0.1	1.4 ± 0.2	7.5 ± 1.4	1.1 ± 0.1	0.6 ± 0.2	10.8 ± 3.6	286 ± 12	44.8 ± 8.1
A^b^	5	0.8 ± 0.2	3.6 ± 0.1	59.1 ± 4.2	4.4 ± 0.2	1.4 ± 0.1	15.8 ± 1.9	793 ± 26	44.3 ± 1.9
B^c^		0.4 ± 0.1	3.4 ± 0.2	37.6 ± 0.4	2.9 ± 0.1	1 ± 0.1	15.1 ± 1.8	584 ± 25	58.2 ± 2.9
C^d^		0.7 ± 0.1	4.9 ± 0.2	50.2 ± 3.8	5.1 ± 0.2	1.7 ± 0.1	12.8 ± 1.2	941 ± 53	87.9 ± 7.4
D^e^		0.2 ± 0.2	3.3 ± 0.3	51.1 ± 3.7	3.6 ± 0.2	0.9 ± 0.1	14.5 ± 1.1	670 ± 55	76.3 ± 1.8
A^b^	6	2.3 ± 0.1	6.9 ± 0.2	39.6 ± 5	4.2 ± 0.3	1.8 ± 0	5.9 ± 4.2	941 ± 36	36.8 ± 1.3
B^c^		0.6 ± 0.1	5.9 ± 0.7	40 ± 0.5	3.5 ± 0.2	1.1 ± 0.1	5.8 ± 4.1	841 ± 48	35.8 ± 0.8
C^d^		0.4 ± 0.2	8.2 ± 0.3	65.8 ± 3	5.7 ± 0.3	2.6 ± 0.3	2.7 ± 1.8	1294 ± 69	36.6 ± 5.2
D^e^		1.2 ± 0.4	5.9 ± 0.9	49.4 ± 5.1	4.4 ± 0.6	1.6 ± 0.2	3.1 ± 2.4	1002 ± 106	40.9 ± 2.2
A^b^	7	0.8 ± 0.2	8.6 ± 0.8	50.5 ± 2.3	5.3 ± 0.2	2.6 ± 0.2	7.8 ± 0.8	1237 ± 40	31.7 ± 2.2
B^c^		2 ± 0.5	8.9 ± 0.9	37.1 ± 2.7	4.4 ± 0.4	1.8 ± 0.4	4.7 ± 3.3	1057 ± 66	30.9 ± 1.3
C^d^		1.4 ± 0.1	10.9 ± 0.8	53.7 ± 5.2	5.9 ± 0.4	2.6 ± 0.5	7 ± 5	1367 ± 74	39.2 ± 1.5
D^e^		1 ± 0.5	10 ± 0.7	46.3 ± 0.9	5.8 ± 0.1	2.9 ± 0.3	9.3 ± 0.8	1360 ± 51	39.3 ± 0.8
A^b^	8	0.8 ± 0	13.8 ± 1.2	47.4 ± 3.6	5.5 ± 0.6	3.3 ± 0.4	12.1 ± 2.7	1400 ± 110	30.1 ± 1.4
B^c^		0.5 ± 0.2	12.1 ± 0.4	38.6 ± 1.2	4.6 ± 0.2	2 ± 0.3	3.1 ± 2.3	1094 ± 55	32.4 ± 2
C^d^		1.1 ± 0.9	4.6 ± 4.3	35.9 ± 1.9	6.4 ± 0.2	3.4 ± 0.3	24.8 ± 2.8	1615 ± 52	33.3 ± 1.6
D^e^		1.5 ± 1.1	4.4 ± 3.9	40.1 ± 0.9	6.1 ± 0.2	2.8 ± 0.1	17.4 ± 0.7	1459 ± 49	33.8 ± 2.3
A^b^	9	2.2 ± 0.6	13.5 ± 1.2	33.4 ± 3.1	5.4 ± 0.2	4.3 ± 0.5	10.9 ± 0.4	1402 ± 40	25.3 ± 0.8
B^c^		4 ± 0.8	8.6 ± 6	18 ± 0.4	3.9 ± 0	2.4 ± 1.7	10.5 ± 1.1	1193 ± 46	20.9 ± 1
C^d^		1.8 ± 0.6	4.7 ± 3.9	28.6 ± 0.6	6.1 ± 0.3	3.8 ± 0.4	19.8 ± 0.8	1595 ± 61	28.1 ± 0.8
D^e^		1.5 ± 0.8	5.5 ± 4.2	31.7 ± 5	5.7 ± 0.6	3.9 ± 0.6	20.6 ± 1.9	1553 ± 153	29.3 ± 0.5
A^b^	10	4.6 ± 1	17.7 ± 1	28.3 ± 2.4	6 ± 0.1	5.1 ± 0.5	16.9 ± 3.5	1570 ± 27	23.9 ± 1.2
B^c^		2.2 ± 0.6	4.9 ± 4.6	25.2 ± 1	4.8 ± 0.2	3.6 ± 0.3	26.1 ± 7.5	1390 ± 79	28.6 ± 4.8
C^d^		1.6 ± 0.4	4.5 ± 3.7	34.3 ± 0.2	6.9 ± 0.3	5 ± 0.3	35.2 ± 2.9	1822 ± 37	33.4 ± 1.3
D^e^		1.3 ± 0.5	10.3 ± 7	37.2 ± 2.1	6.9 ± 0.3	5.3 ± 0.4	35.1 ± 2.8	1915 ± 39	36.2 ± 2.5
A^b^	11	1.2 ± 0.4	5.2 ± 4.6	41.8 ± 2.7	6.6 ± 0.7	6.3 ± 0.8	38.2 ± 2.8	1816 ± 158	33.2 ± 1.6
B^c^		0.6 ± 0.4	15.2 ± 2.6	34.8 ± 5.5	5.5 ± 0.9	4.4 ± 0.4	20 ± 2.9	1599 ± 184	30 ± 4.2
C^d^		1.3 ± 0.3	0.6 ± 0.2	32.8 ± 0.8	6.8 ± 0.3	5.6 ± 0.3	41.3 ± 1.6	1894 ± 75	35.8 ± 6.5
D^e^		0.8 ± 0.3	13.2 ± 1.2	36 ± 1.3	5.4 ± 0.5	4.4 ± 0.3	18.8 ± 0.8	1541 ± 126	28.4 ± 2
A^b^	12	0.8 ± 0.2	19.6 ± 4.3	34.2 ± 4.9	5.8 ± 0.9	5.4 ± 0.9	30.9 ± 9.4	1605 ± 238	31.7 ± 3.7
B^c^		0.5 ± 0	11.9 ± 0.2	27.9 ± 1.5	4.5 ± 0.3	3.9 ± 0.3	24.9 ± 2.7	1309 ± 39	23.6 ± 1.1
C^d^		0.6 ± 0.1	11.5 ± 0.4	30.6 ± 1.3	5.2 ± 0.3	5.2 ± 0.7	20.8 ± 5.8	1473 ± 55	29.8 ± 1.4
D^e^		0.7 ± 0.1	13.3 ± 0.7	37.6 ± 2.2	5.9 ± 0.4	5.9 ± 0.2	22.2 ± 1.3	1672 ± 84	29.8 ± 1
A^b^	13	1.5 ± 0.1	13.4 ± 0.8	35.3 ± 1.9	5.6 ± 0.5	5 ± 0.2	21.1 ± 2.2	1540 ± 119	28.9 ± 1.6
B^c^		1.4 ± 0.4	12.7 ± 0.2	30.4 ± 3.3	4.3 ± 0.6	3.9 ± 0.4	22 ± 1	1305 ± 137	29.6 ± 7.4
C^d^		1.6 ± 0.4	12.5 ± 1.2	36.7 ± 4.1	5.9 ± 0.6	5.9 ± 0.6	23.1 ± 4	1636 ± 157	41.8 ± 9.5
D^e^		1 ± 0.2	10.9 ± 0.2	34.3 ± 4.6	4.8 ± 0.4	4.5 ± 0.8	18.2 ± 1.7	1387 ± 107	27.1 ± 1.3

CBDVA, cannabidivarinic acid; CBDA; cannabidiolic acid; CBGA, cannabigerolic acid; THCVA, tetrahydrocannabivarinic acid; CBNA, cannabinolic acid; Δ^9^-THC, Δ^9^-tetrahydrocannabinol; THCA, tetrahydrocannabinolic acid; and CBCA, cannabichromenic acid.

^a^All, control and enhanced treatments; ^b^A, control treatment; ^c^B, enhanced treatment with the addition of amino acids; ^d^C, enhanced treatment with the addition of P, K, and Fe; ^e^D, enhanced treatment with the addition of amino acids, P, K, and Fe.

### Antibacterial activity of cannabis extracts

The susceptibility of *S. aureus* strains tested in our study was confirmed by control with oxacillin. The minimum inhibitory concentration (MIC) of oxacillin for *S. aureus* ATCC 29213 was 0.25 µg/mL, and for ATCC 43300 it was 32 µg/mL. Our findings show no remarkable differences between values of MIC observed in this study and sensitivity of *S. aureus* ATCC 29213 (MIC 0.12–0.5 µg/mL) as well as the resistance of *S. aureus* ATCC 43300 (MIC > 4 µg/mL) interpreted by Clinical and Laboratory Standards Institute (CLSI)^47^. All the tested cannabis extracts had a growth-inhibitory effect on each *S. aureus* strain. The strongest antibacterial activity was observed from the 5th to the 13th week of *C. sativa* growth, when the MICs reached values ranging from 32 to 64 μg/mL. In comparison, extracts from the 1st to the 4th week produced moderate activity against the *S. aureus* strains (MICs 128–256 µg/mL). In this study, no statistically significant differences were found between the susceptibility of MSSA and MRSA or between individual nutritional treatments. The susceptibilities of both tested *S. aureus* strains to the cannabis extracts across all vegetation stages (weeks 1–13) are presented in Table 2.

**Table 2:** Minimum inhibitory concentrations (µg/mL) of ethanolic cannabis extracts against *Staphylococcus aureus* strains.

Treatment	Week/*Staphylococcus aureus* ATCC strain/MIC (µg/mL)												
	1	2	3	4	5	6	7	8	9	10	11	12	13
29213													
A^a^	256	128	256	256	64	64	32	32	32	32	64	64	32
B^b^	256	128	256	256	64	64	32	32	64	32	64	64	64
C^c^	256	256	256	256	64	32	32	32	32	32	32	32	32
D^d^	256	256	256	256	64	64	32	32	32	32	32	32	32
43300													
A^a^	256	128	128	128	64	64	32	32	32	32	32	32	32
B^b^	256	128	128	256	64	64	32	32	32	32	32	32	64
C^c^	256	256	256	256	64	32	32	32	32	32	32	32	32
D^d^	256	256	256	256	64	64	32	32	32	32	32	32	32

^a^A, control treatment; ^b^B, enhanced treatment with the addition of amino acids; ^c^C, enhanced treatment with the addition of P, K and Fe; ^d^D, enhanced treatment with the addition of amino acids, P, K and Fe.

### Statistical evaluation of the dependence of growth inhibition on individual phytocannabinoids

Statistical analyses based on stepwise regression models of the dependence of the MIC on *S. aureus*, ATCC 29213 and 43300 strains and on the concentrations of individual cannabinoids in cannabis extracts showed that THCVA was the most significant of all eight models and alone contributed to adjusted R-squared values of 0.743 and above. After stepwise selection, more variables were added to the models. However, there was no consistent selection across the eight models, and the variables seemed to be chosen more or less at random. This can probably be explained by the data having 13 rows and 13 additional columns to pick (i.e., three columns/phytocannabinoids were removed from the initial 17 columns/phytocannabinoids, THCVA was used as the primary independent variable, and 13 columns/phytocannabinoids were left to work with). There was a high likelihood that at least some columns/phytocannabinoids could enter the model and reduce error, improving its accuracy. Thus, apart from the THCVA column, the inclusion of other variables was not considered to indicate statistical significance. The risk of an artifactual model improvement was very high, as more data supplied into the model increased the odds of finding a variable improving the model without a corresponding real dependence.

A summary of the stepwise regression models is shown in Table 3. Considering that the MIC values for *S. aureus* strains ATCC 29213 and ATCC 43300 were the same after the mode was applied for the repeated measurements, the four treatment regimens yielded the same results for both strains. The table shows only the four nonduplicated results for the adjusted R-squared values of the created models. The first represents the adjusted R-squared value of a ‘simple’ model, where the only independent variable was THCVA. The second number is the adjusted R-squared value of a ‘full’ model, where stepwise selection (with a threshold *p* value of 0.15) was used to include or remove more variables. After the stepwise selection was complete, the remaining independent variables left in each model were displayed in the last column of the table.

**Table 3:** Summary of results of the stepwise regression models.

Treatments	Model adjusted R-squared^e^	Model adjusted R-squared^f^	Independent variables^g^
A^a^	0.788	0.863	THCVA, Δ^9^-THC
B^b^	0.743	0.945	THCVA, CBLA, CBC, CBNA
C^c^	0.962	0.989	THCVA, CBDA, CBDVA
D^d^	0.901	0.972	THCVA, CBGA, THCA

CBC, cannabichromene; CBDVA, cannabidivarinic acid; CBDA; cannabidiolic acid; CBGA, cannabigerolic acid; CBNA, cannabinolic acid; CBLA, cannabicyclolic acid; Δ^9^-THC, Δ^9^-tetrahydrocannabinol; THCA, tetrahydrocannabinolic acid; THCVA, tetrahydrocannabivarinic acid.

^a^A, control treatment; ^b^B, enhanced treatment with the addition of amino acids; ^c^C, enhanced treatment with the addition of P, K, and Fe; ^d^D, enhanced treatments with the addition of amino acids, P, K, and Fe. ^e^Only THCVA included. ^f^Full stepwise selection. ^g^Included in the full stepwise model.

## Discussion

Our findings show no remarkable differences in the observed MIC values of oxacillin, used as a positive control in this study, compared to the sensitivity of *S. aureus* ATCC 29213 (MIC 0.12–0.5 µg/mL) as well as the resistance of *S. aureus* ATCC 43300 (MIC > 4 µg/mL) interpreted by CLSI^47^. In the context of our research, all the ethanolic extracts from cannabis plants inhibited the growth of both *S. aureus* strains in vitro (MIC 32–256 µg/mL), including antibiotic-resistant and antibiotic-sensitive forms, across all vegetation stages (weeks 1–13) under the various nutritional treatments. According to recent studies, crude cannabis extracts have demonstrated growth-inhibitory effects against staphylococcal strains over wide ranges of MIC values, from 4 to 8000 µg/mL^48–51^. These findings correspond partly with the results of our experiments. The small variations between our results and previously published data can be explained by the different methodologies and bacterial strains used. For example, Skala et al.^48^ and Giselle et al.^49^ reported MIC values of 8 and 115.25 µg/mL, respectively, for ethanolic cannabis extracts, measured by the microdilution broth method, against *S. aureus* (ATCC 25923). Kaur et al.^50^ determined that the MIC of a methanolic extract of *C. sativa* against *S. aureus* ATCC 25923 was 1560 µg/mL using the agar-well diffusion method. To the best of our knowledge, this is the first study demonstrating the antibacterial effect of ethanolic cannabis extracts across the whole vegetation cycle in a wide range of nutritional interventions.

The HPLC–DAD results showed that the most concentrated cannabinoid in the plants and consequently in the extracts was THCA. The statistical analysis revealed that a homolog of THCA, THCVA, influenced the antibacterial activity of the cannabis extracts against both strains tested by approximately 74.3%. Among the group of cannabinoids, the most active antimicrobial compounds reported were THC, CBD, and CBG^52,53^. THCVA is produced in very low quantities by most cannabis cultivars and, for that reason, has been little studied. Several reports have suggested that THCVA is produced by a variant of the well-known cannabinoid pathway that produces homologous compounds such as cannabigerovarinic acid (CBGVA), which is the precursor molecule used by THCVA and CBDVA synthases^54,55^. In general, phytocannabinoids contain a monoterpene unit attached to a phenolic ring containing a C3 alkylated carbon^56^. The alkyl side chain can vary from one to seven carbons, but *n*-pentyl is the most abundant^57^. Phytocannabinoids containing an *n*-propyl side chain are called cannabivarins. THCVA a type of phytocannabinoid that occurs primarily in the form of acids, is often found in *C. sativa* subsp. *indica* and refers to the ‘narrow-leaflet drug biotype’, for which the cultivar THCVA is the chemotaxonomic marker^58^.

It has previously been documented that phytocannabinoids, especially CBD, exhibit membrane-related activity, causing depolarization of the cytoplasmic membrane and disruption of the mitochondrial membrane potential in *S. aureus*^59^. Appendino et al.^60^ concluded that the resorcinol moiety of phytocannabinoids serves as an antibacterial pharmacophore, with alkyl, terpenoid, and carboxylic groups modulating the activity. These functional groups make phytocannabinoids highly hydrophobic compounds^61^. Gram-positive bacteria have a thick layer of peptidoglycan linked to other hydrophobic molecules, such as proteins and teichoic acid^62^. Therefore, it is possible to assume that this hydrophobic layer surrounding gram-positive bacterial cells may facilitate easy entry of hydrophobic molecules^63^. Interestingly, THCVA is structurally similar to THC except for the presence of a shortened side chain (a propyl side chain instead of a pentyl)^64^ and a free carboxyl group^57^. Generally, carboxylic acids can easily release protons^65^, which can lead to damage of the cell membrane and a decrease in the internal pH^66^. This reaction can negatively affect enzymatic reactions and metabolic pathway in bacteria^67^. Based on these findings, it is possible to assume that the key factor in the antimicrobial mechanism of phytocannabinoids is their chemical structure. However, further research focused on a better understanding of the antimicrobial mechanism of THCVA is warranted.

In summary, the present study demonstrated the antistaphylococcal activity of ethanolic extracts of *C. sativa* L. against both of the bacterial strains tested, MSSA and MRSA, across all the vegetation stages, especially from the 5th to the 13th week. The various nutritional treatments had no impact on the resulting antibacterial effect. However, from a statistical point of view, these findings contributed to the more significant heterogeneity of the cannabis extracts and subsequent proof of their possible effect. For the first time, this study identified THCVA as the main cannabinoid responsible for antibacterial activity. From the point of view of future studies, it would be interesting to use a cannabis hybrid genotype with a greater representation of *C. sativa* subsp. *indica* or one with the chemical phenotype of cannabinoid THCVA dominance. However, further studies on its in vivo antibacterial activity and safety are needed before clinical trials can be performed. Furthermore, investigations concerning the exact mechanism of the antimicrobial effects of THCVA should be conducted.

## Methods

### Cultivation of cannabis plants

The soilless cultivation of cannabis plants in expanded clay was conducted in a room with controlled lighting, temperature, and humidity. For the experiments, vegetative propagation of clones was performed by cutting from apical positions with at least three fully expanded uncut leaves obtained from *C. sativa* L. ‘McLove’ mother plants^68^. This cultivar is characterized by its elevated THC content, reaching up to 20%, while its CBD levels remain below 0.3%. The cannabinoid profile measurement classifies it as chemotype I, indicating a high THCA/CBDA ratio, which exceeds 1^69^. The cuttings were rooted in rock-wool cubes (4 × 4 cm) under light-emitting diodes for approximately 3 weeks. Rooted clones were then transferred to polypropylene pots with a volume of 3.45 L filled with 3 L of expanded clay (EuroPebbles) on the growing tables. Each cultivation table measured 2 m^2^ (1 × 2 m) and represented a different treatment with an independent 100 L nutrient solution tank. The plant density was 27.5 per m^2^ (55 plants/table/treatment). Capillaries provided drip irrigation on a timer set for nine irrigation cycles, each lasting 60 s, all of which occurred during the daylight phase. During one irrigation cycle, 94 mL of the nutrient solution was supplied to each plant for a total of 846 mL per plant per day. Six high-pressure sodium lamps, each rated at 1000 W, provided a suitable light spectrum with a photosynthetic photon flux density (PPFD) of 1029 μmol/m^2^/s. The light regimen was set to 18 h of light and 6 h of darkness for the first week during the vegetative (growth) phase. The light phase temperature was kept at 25 °C and was reduced to 22 °C during the dark phase. The relative humidity was maintained at 60%, and the CO_2_ concentration was 1065 mg/m^3^ (1.065 mg/L). After the second week, the cultivation regimen was adjusted to the generative (flowering) phase. The photoperiod was set to 12 h of light and 12 h of darkness. The CO_2_ concentration and temperature were kept the same as those in the vegetative phase, but the relative humidity was reduced to 40% to lower the risk of fungal growth.

### Hydroponic nutrient treatments

The plants were cultivated in the soilless cultivation system and subjected to three enhanced nutritional treatments compared to the control treatment (A). A fresh nutrient solution was prepared from water (DMW) demineralized by reverse osmosis every 7 days, starting from the first day of the experiment. The nutrient solution was recirculated for 1 week without the addition of other nutrients, and the pH was checked and adjusted daily to 5.9^70^. The first enhanced treatment (B) involved the addition of an amino acid biostimulant (composition previously described)^71^ from the second week for the last 24 h at a volume of 2 mL/L before the nutrient solution was changed. Subsequent enhanced treatment (C) increased the amounts of P (P_2_O_5_), K (K_2_O), and Fe (chelated) added beginning in the 5th week. The third enhanced treatment (D) was a combination of the two enhanced treatments, B and C. The nutrient content increased according to the age of the plants. Beginning in the 10th week, the plants were irrigated only with DMW. The electrical conductivity (EC) of the new solution from each nutritional treatment was recorded during mixing, and samples were collected for analysis every week. The measured nutrient compositions of the fresh solutions are shown in Table 4^72^.

**Table 4.** Nutrient content in control (A) and enhanced treatment (B, C, D) solutions (mg/L).

Treatment	Elements	Weeks						
		1	2	3	4	5	6–9	10–13
A^a^, C^c^	N	100 ± 1	115 ± 1	129 ± 2	150 ± 2	129 ± 2	150 ± 2	DMW^f^
B^b^, D^d^			302 ± 2	333 ± 3	352 ± 3	332 ± 3	353 ± 3	DMW^f^
A^a^, B^b^	P	32.2 ± 0.5	39.9 ± 0.6	44.0 ± 0.8	51.9 ± 0.6	44.0 ± 0.8	51.9 ± 0.7	DMW^f^
C^c^, D^d^						92.0 ± 1.8	92.9 ± 1.8	DMW^f^
A^a^, B^b^	K	125 ± 2	151 ± 1	174 ± 2	194 ± 2	174 ± 2	194 ± 2	DMW^f^
C^c^, D^d^						258 ± 2	266 ± 3	DMW^f^
All^e^	Ca	98.3 ± 1.2	120 ± 2	133 ± 2	147 ± 2	133 ± 1	144 ± 2	DMW^f^
All^e^	Mg	25.3 ± 0.3	30.9 ± 0.3	34.0 ± 0.5	40.0 ± 0.4	33.0 ± 0.4	38.6 ± 0.6	DMW^f^
A^a^, C^c^	S	21.5 ± 0.2	26.0 ± 0.9	30.9 ± 0.4	33.7 ± 0.3	30.8 ± 0.4	34.9 ± 0.3	DMW^f^
B^b^, D^d^			51.1 ± 0.6	56.0 ± 0.5	62.0 ± 0.9	55.9 ± 0.6	62.0 ± 0.8	DMW^f^
A^a^, B^b^	Fe	0.92 ± 0.10	1.13 ± 0.08	1.20 ± 0.09	1.47 ± 0.08	1.20 ± 0.10	1.46 ± 0.08	DMW^f^
C^c^, D^d^						12.2 ± 0.3	13.7 ± 0.9	DMW^f^
All^e^	Mn	0.65 ± 0.05	0.74 ± 0.04	0.80 ± 0.06	1.00 ± 0.08	0.76 ± 0.07	0.94 ± 0.09	DMW^f^
All^e^	Zn	0.21 ± 0.05	0.28 ± 0.01	0.28 ± 0.03	0.35 ± 0.04	0.29 ± 0.05	0.33 ± 0.05	DMW^f^
All^e^	Cu	0.07 ± 0.01	0.08 ± 0.01	0.10 ± 0.01	0.13 ± 0.01	0.11 ± 0.02	0.11 ± 0.01	DMW^f^
All^e^	B	0.16 ± 0.01	0.19 ± 0.02	0.21 ± 0.01	0.25 ± 0.02	0.22 ± 0.02	0.24 ± 0.02	DMW^f^
All^e^	Mo	0.01 ± 0.00	0.02 ± 0.00	0.02 ± 0.00	0.03 ± 0.00	0.02 ± 0.01	0.02 ± 0.01	DMW^f^
A^a^	EC	0.97 ± 0.01	1.20 ± 0.01	1.46 ± 0.01	1.74 ± 0.01	1.46 ± 0.01	1.74 ± 0.01	DMW^f^
C^c^						2.05 ± 0.02	2.34 ± 0.06	DMW^f^
B^b^			1.38 ± 0.01	1.71 ± 0.01	2.14 ± 0.01	1.71 ± 0.01	2.14 ± 0.01	DMW^f^
D^d^						2.30 ± 0.02	2.74 ± 0.02	DMW^f^

^a^A, control treatment; ^b^B, enhanced treatment with addition of amino acids; ^c^C, enhanced treatment with addition of P, K, and Fe; ^d^D, enhanced treatments with the addition of amino acids, P, K, and Fe; ^e^all, control and enhanced treatments. ^f^DMW, demineralized water.

### Plant sampling and drying

Three replicates of the plants were harvested weekly from each treatment group throughout the vegetation cycle. The entire aboveground biomass of each plant was clipped and subsequently divided into stems, leaves, and flowers. The flowers were dried to constant moisture (8–10%) at 25 °C. A reference amount of each flower sample was dried at 105 °C until it reached a stable weight, after which the total moisture content was determined. Subsequently, the dried flowers (including leaves up to the 4th week) were frozen in liquid nitrogen and ground.

### Extraction and measurement of phytocannabinoids

Cannabinoids from homogenized ground flowers (including leaves up to the 4th week) were extracted by an optimized dynamic maceration method^73^. Weighed samples (0.3 g) were mixed with 10 mL of 96% ethanol and macerated for 1 h at room temperature under constant magnetic stirring at 300 rpm. Subsequently, the mixtures were filtered under vacuum using a Morton filter device (porosity S4/P16), and the filtrates were collected. The flowers were removed from the filter and extracted twice more with 10 mL of ethanol, after which the filtrates were combined. Aliquots of 0.5 mL of each sample were diluted with 96% ethanol to a volume of 10 mL and filtered through a 0.22 μm nylon syringe filter into a vial. The extracted samples were injected into a high-performance liquid chromatography system equipped with a diode array detector (HPLC–DAD; Agilent 1260, Agilent Technologies, Inc., USA) and a 250 × 3 mm Luna® C18 Column (2) with a particle size of 3 µm (Phenomenex, USA). The isocratic mobile phase consisted of acetonitrile/water (31:9, v/v) with 0.1% formic acid (v/v) and 0.1 M ammonium formate (without pH adjustment). The flow rate was 0.55 mL/min, and the temperature was 37 °C. The sample injection volume was 8 μL, and UV detection was performed at 275 nm^74^. The instrument was externally calibrated using 0.16 to 100 mg/L THCA and 0.16–10 mg/L other phytocannabinoids (Sigma‒Aldrich, Czech Republic) as standards. The data were analyzed with OpenLAB CDS (ChemStation Edition, rev. C.01.5).

### Bacterial strains and growth medium

The standard *S. aureus* strains ATCC 29213 (MSSA) and ATCC 43300 (MRSA) were obtained from the American Type Culture Collection (ATCC, Rockville, MD, USA). Cation-adjusted Mueller–Hinton broth (Oxoid, Basingstoke, UK) was used as the cultivation and assay medium for both *S. aureus* strains.

### Determination of the minimum inhibitory concentration

The MICs were determined using the standard broth microdilution method in 96-well microtiter plates according to approved guidelines and recommendations for susceptibility testing of aerobic bacteria^47^, with the modifications proposed by Cos et al.^75^ for more effective assessment of the anti-infective potential of natural products. Serial dilutions of the extracts were prepared in an appropriate growth medium (90 µL), ranging from 4 to 512 µg/mL, using a manual multichannel pipette (Eppendorf, Wesseling-Berzdorf, Germany). The plates were inoculated with a bacterial suspension at a final density of 5 × 10^5^ CFU/mL using the McFarland scale and incubated at 37 °C for 24 h under aerobic conditions. Bacterial growth was subsequently assessed by turbidity determination via an Infinite 200 PRO microplate reader (Tecan, Männedorf, Switzerland) at 405 nm according to Cos et al.^75^. Oxacillin (Sigma‒Aldrich, Prague, Czech Republic) was dissolved in distilled water and used as a positive control. All tests were performed as three independent experiments, each carried out in triplicate, and the results are presented as modal values.

### Statistical evaluation of the dependence of growth inhibition on specific phytocannabinoids

The data were prepared using a custom-made Python script that produced four MS Excel files, one for each treatment (A, B, C, and D). Each file contained a table with 19 columns, 2 columns for the dependent variables (MICs of cannabis extracts against *S. aureus* strains, ATCC 29213 and 43300) and 17 columns for the independent variables representing the contents of the examined phytocannabinoids in the extracts. The size of the tables was 19 × 13 (13 weeks of vegetation). The values of the dependent variable were calculated as modes from repeated MIC measurements. The values for the independent variables were the average concentrations of the individual phytocannabinoids in the extracts. The data were statistically evaluated using a script in R-Studio software and the following libraries: ggplot2, dplyr, GGally, caret, and olsrr. The independent variable columns were first trimmed using the nearZeroVar function. As a result, three columns with the phytocannabinoids THCV, cannabinol (CBN), and cannabicyclol (CBL), which contained mostly zero values, were removed; the overall variance in these columns was very low. Two linear models were then created, one for each dependent variable. The ‘lm’ and ‘ols_step_both_p’ functions were combined to construct eight stepwise regression models.

All the experimental research, including the collection of plant material, was performed in accordance with the relevant national or international guidelines/regulations/legislation.

## Data Availability

The datasets used and/or analysed during the current study available from the corresponding author on reasonable request.
